# The complete mitochondrial genome of the *Montipora peltiformi* (Scleractinia: Acroporidae)

**DOI:** 10.1080/23802359.2017.1419100

**Published:** 2018-01-08

**Authors:** Xin Wang, Peng Tian, Wentao Niu, Shuangen Yu

**Affiliations:** a School of Marine Sciences, Forestry College, Guangxi University, Nanning, China;; b Guangxi Key Lab of Mangrove Conservation and Utilization, Guangxi Mangrove Research Center, Guangxi Sciences Academy, Beihai, China;; c Laboratory of Marine Biology and Ecology, Third Institute of Oceanography, State Oceanic Administration, Xiamen, China

**Keywords:** Coral, mitogenome, phylogeny

## Abstract

In this study, the complete mitogenome sequence of stony coral, *Montipora peltiformi* (Scleractinia), has been decoded for the first time by next generation sequencing and genome assembly. The assembled mitogenome, consisting of 17,884 bp, has unique 13 protein coding genes (PCGs), three transfer RNAs, and two ribosomal RNAs genes. The complete mitogenome of *Montipora peltiformi* showing 99% identities to *Montipora cactus*. The complete mitogenome provides essential and important DNA molecular data for further phylogenetic and evolutionary analysis for coral phylogeny.

Reef-building corals play an important role in shallow tropical seas by providing an environmental base for the ecosystem. The Acroporidae is one of the most important families of reef corals, they dominate the major reef assemblages in the Indo-Pacific. The family Acroporidae consists of four genera, Acropora, Montipora, Anacropora, and Astreopora (Fukami et al. 2000). *Montipora peltiformi*, one of the members of Acroporidae family, is widely distributed throughout the Indo-Pacific. And it is common in most reef environments. The first establishment of *Montipora peltiformi* mitogenome is important for further evolutionary and phylogenetic analyses for stony coral (Tseng et al. 2005).

Samples (voucher no. DYW19) of *Montipora peltiformi* were collected from Daya Bay in Guangdong, China. We used next generation sequencing to perform low-coverage whole genome sequencing according to the protocol (Niu et al. 2016). Initially, the raw next generation sequencing reads generated from HiSeq 2000 (Illumina, San Diego, CA). About 0.2% raw reads (32,752 out of 16,549,722) were *de novo* assembly by using commercial software (Geneious V9, Auckland, New Zealand) to produce a single, circular form of complete mitogenome with about an average 458 × coverage.

The complete mitogenome of *Montipora peltiformi* was 17,884 bp in size (GenBank KY094487) and its overall base composition is 24.9% for A, 14.18% for C, 24.13% for G, and 36.794% for T, and have GC content of 38.31%, showing 99% identities to *Montipora cactus* (GenBank AY903296.2). The protein coding, rRNA and tRNA genes of *Montipora peltiformi* mitogenome were predicted by using DOGMA (Wyman et al. 2004), ARWEN (Laslett and Canback 2008), MITOS (Bernt et al. 2013) tools, and manually inspected. The complete mitogenome of *Montipora peltiformi* includes unique 13 protein-coding genes (PCGs), three transfer RNA genes (tRNA-Met, tRNA-Arg, tRNA-Trp), and two ribosomal RNA genes. All PCGs, tRNA and rRNA genes were encoded on H-strand. The PCG of NAD5 has a 11,489bp intron insertion. It is important to note that two PCGs started with ATG codon (ATP6 and ATP8), one with ATT codon (ND5), one with GTG codon (ND4L), two with ATA codon (ND6 and COX2), and seven with TTA codon (ND1, Cyt b, ND2, ND4, COX3, COX1, and ND3). Six of the 13 PCGs are inferred to terminate with TAA (ND1, ND2, ND6, COX2, ND4L, and COX1), six PCGs with TAG (Cyt b, ATP6, ND4, ND3, ND5, and ATP8), one with TCA (COX3). Among the 13 PCGs, the longest one is ND5 gene (1686 bp), whereas the shortest is ATP8 gene (219 bp). There were 7bp overlapping nucleotides between ND4L and tRNA-Arg, 10bp overlapping nucleotides between ATP8 and COX1, and the number of non-coding nucleotides between different genes varied from 8 to 738 bp.

To validate the phylogenetic position of *Montipora peltiformi,* we used MEGA7 software (Kumar et al. 2016) to construct a Maximum likelihood tree (with 500 bootstrap replicates and Kimura 2-parameter model) containing complete mitogenomes of 10 species derived from Astrocoeiina of Scleractinia. *Turbinaria peltata derived from Dendrophylliidae* was used as outgroup for tree rooting. Result shows *Montipora peltiformi* is closely related to *Montipora cactus* with high bootstrap value supported (Figure 1). In conclusion, the complete mitogenome of the *Montipora peltiformi* deduced in this study provides essential and important DNA molecular data for further phylogenetic and evolutionary analysis for stony coral phylogeny.

**Figure 1.**
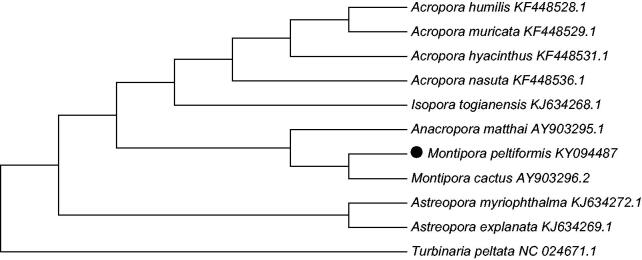
Molecular phylogeny of *Montipora peltiformi* and related species in Scleractinia based on complete mitogenome. The complete mitogenomes is downloaded from GenBank and the phylogenic tree is constructed by Maximum likelihood method with 500 bootstrap replicates. The gene’s accession number for tree construction is listed behind the species name.
